# Editorial: The Involvement of NGF in the Alzheimer's Pathology

**DOI:** 10.3389/fnins.2019.00872

**Published:** 2019-08-28

**Authors:** A. Claudio Cuello

**Affiliations:** Charles E. Frosst/Merck Chair in Pharmacology, McGill University, Montreal, QC, Canada

**Keywords:** nerve growth factor, Alzheimer's disease pathology, basal forebrain cholinergic neurons, neurodegeneration, trophic factors

Since the historical and game-changing discovery of NGF by the late Rita Levi-Montalcini (Levi-Montalcini et al., 1996; Levi-Montalcini, 2004) a myriad of publications have emerged on this growth factor. Further to it, the neurotrophic family was extended and newer trophic factors were discovered. Trophic factors were found responsible for synaptic patterning, developmental survival of specific neuronal systems and neuronal-synaptic phenotypic maintenance in adulthood. Many of the fundamental discoveries related above were made by authors of this Special Issue of Frontiers.

The role of NGF in the pathology of Alzheimer's disease (AD) has been of great interest in the last decades. Soon after its discovery, it was demonstrated that NGF played an essential role on the survival of forebrain cholinergic neurons during developmental stages and subsequently a role on the maintenance of their cholinergic phenotype at neuronal cell-somata and synaptic levels. Since this cholinergic system was shown to be one of the first casualties of the Alzheimer's pathology, great research interest was placed on this relationship. This was much more the case since the forebrain cholinergic system plays an important role in higher CNS functions like attention, memory, and learning.

This set of neurons is likely the most NGF-dependent of the entire adult nervous system. In early years, there was a strong interest in assessing the potential value of applying exogenous NGF for the recovery of experimentally damaged cholinergic septo-hippocampal and basalo-cortical neuronal pathways. The NGF capability to prevent and even to recover these degenerating pathways was shown to be remarkable and an exciting novelty at the time. Such effects provoked an interest in the possible application of this principle in AD, an aspect which still remains alive and well-represented in this collection of NGF-AD related contributions.

The participation of NGF in a wide range of aspects of the AD pathology poses a number of significant biological questions. The field of research continues to attract individual scientists and research groups alike. For this special issue of Frontiers we convened a number of outstanding investigators for the purpose of assembling a diversity of views related to outstanding questions such as: what is the role of NGF in the continuum of the AD pathology?, what is its involvement on the atrophy of the basal forebrain neurons?, and what are the translational possibilities of such a body of knowledge?

I am most gratified that very distinguished colleagues responded positively to the call of generating significant revisions. Further to it, I would think that this Special Issue of Frontiers will be of value to many and that it will inspire new and transformative research on NGF in the realm of the AD pathology.

## Author Contributions

The author confirms being the sole contributor of this work and has approved it for publication.

### Conflict of Interest Statement

The author declares that the research was conducted in the absence of any commercial or financial relationships that could be construed as a potential conflict of interest.
